# Occupational tasks associated with shoulder pain and upper extremity disability: a cross-sectional study in the Johnston County Osteoarthritis Project

**DOI:** 10.1186/s12891-024-07487-x

**Published:** 2024-05-11

**Authors:** Elizabeth L. Yanik, Carolina Alvarez, Rebecca J. Cleveland, Amanda E. Nelson, Yvonne M. Golightly

**Affiliations:** 1 Department of Orthopaedic Surgery, Washington University School of Medicine, 660 S. Euclid Ave, St. Louis, MO 63110 USA; 2 Thurston Arthritis Research Center, University of North Carolina, Chapel Hill, NC USA; 3 College of Allied Health Professions, University of Nebraska Medical Center, Omaha, NE USA

**Keywords:** Shoulder, Physical work, Disability

## Abstract

**Background:**

Shoulder pain is a leading cause of disability. Occupations requiring high upper extremity demands may put workers at greater risk of shoulder injury and resulting pain. We examined associations of occupation with shoulder pain and upper extremity disability in the Johnston County Osteoarthritis Project.

**Methods:**

Work industry and occupational tasks for the longest job held were collected from participants. At follow-up ranging from 4–10 years later, participants were asked about shoulder symptoms (pain, aching, or stiffness occurring most days of 1 month in the last year) and given a 9-item, modified Disabilities Arm Shoulder and Hand (DASH) questionnaire to categorize disability from 0–4 (none-worst). Logistic regression and cumulative logit regression models were used to estimate associations with prevalent shoulder symptoms and with worse disability category, respectively. Models were adjusted for cohort, age, sex, race, education and time to follow-up. Sex- and race-stratified associations were evaluated.

**Results:**

Among 1560 included participants, mean age was 62 years (standard deviation ± 9 years); 32% were men, and 31% were Black. Compared to the managerial/professional industry, higher odds of both shoulder symptoms and worse upper extremity disability were seen for most industrial groups with physically demanding jobs, particularly the service industry. Work that often or always required lifting/moving > 10 lbs. was associated with higher odds of shoulder symptoms. Work that sometimes or always required heavy work while standing was associated with higher odds of shoulder symptoms, and this association was stronger among men and White workers.

**Conclusion:**

Physically demanding occupations were associated with increased occurrence of shoulder pain and disability. Mitigating specific physical work demands may reduce shoulder-related disability.

## Introduction

Shoulder injury and resulting pain is a leading cause of disability, with shoulder osteoarthritis (OA) and rotator cuff disease being two of the most frequent causes of shoulder disability. In the US, over 90,000 occupational shoulder injuries occur annually, with shoulder injuries leading to a higher median number of days away from work than any other body part (2015 median = 23 days) (1). Occupations that require high upper extremity demands may put individuals at greater risk of mechanical stresses and may be a key risk factor for shoulder disability.

Studies in European populations have shown that occupational upper extremity loads are associated with shoulder symptoms and risk of shoulder disorders [2–5]. A general population cohort of 883 people in Finland demonstrated associations of repetitive movements, lifting heavy loads, and working in awkward positions with chronic shoulder disorders [2]. In a study of over 30,000 people in Denmark, high occupational shoulder load was associated with significantly higher risk of surgery for subacromial impingement syndrome [3]. Few such studies have been conducted in the United States, where workers may be even more strongly impacted by physical work exposures due to weaker workplace protections as evidenced by higher rates of workplace accidents [6]. Additionally, some analyses have led to inconsistent findings across populations, such as the evaluation of differences in the effects of occupational exposures by sex [2, 7].

We utilized data from the Johnston County Osteoarthritis Project (JoCoOA), a community-based prospective cohort in a mostly rural county in North Carolina that collected data on occupational exposures and measures of shoulder symptoms and upper extremity disability. A prior cross-sectional analysis of JoCoOA demonstrated that 26% of participants reported shoulder symptoms, but associations with occupational demands were not investigated [8]. JoCoOA captures a racially diverse population, as the cohort was developed to have adequate sample sizes to allow evaluation of differences in osteoarthritis development and progression by race. In the United States, physically-demanding jobs are more likely to be done by non-white workers [9], making it imperative to demonstrate that the occupational risks identified in primarily white, European populations generalize to non-white workers.

In the current study, we aimed to determine if occupation industry or occupational tasks involving the upper extremities are associated with prevalent shoulder symptoms or upper extremity disability. We also aimed to determine whether associations differed by race or sex.

## Materials and methods

### Study population

**Fig. 1:**
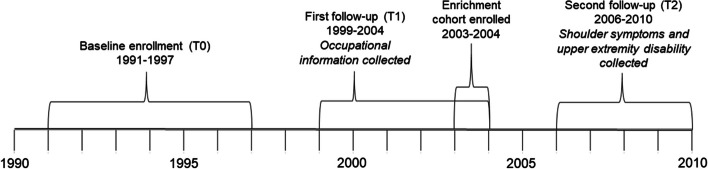
Timeline of enrollment and study visits in the Johnston County Osteoarthritis Project

JoCoOA is a community-based prospective cohort originally established to study knee and hip OA among men and women who identified as Black or White. Participants were recruited from the noninstitutionalized population of adults 45 years of age and older residing in Johnston County, North Carolina. Further details on recruitment methods and sampling strategies have been reported previously [10]. An initial round of enrollment occurred between 1991 and 1997 during which baseline information was collected through home interviews. For these participants a first follow-up visit (T1) was conducted between 1999 and 2004 during which occupational information was collected (N = 1733). New participants were enrolled in the cohort in 2003–2004 with the same occupational information collected at enrollment (N = 1015). The second round of recruitment aimed to enrich the sample for individuals who were Black and younger. We refer to this second group of enrolled participants as the enrichment cohort. A second follow-up visit (T2) was conducted during 2006–2010 at which information on shoulder symptoms and upper extremity disability were collected (Fig. 1).

The study population for the present analysis included participants who completed both T1 and T2 follow-up visits (N = 1697). From this population, we excluded: 1) participants who did not respond to any of the questions about occupation at T1, 2) participants who did not respond to questions about shoulder symptoms or upper extremity disability, and 3) a small fraction of participants missing information on BMI or education (other demographic information was complete). With this population, we conducted a cross-sectional analysis of associations between occupational measures and shoulder disability measures.

### Occupational exposures

Occupational information was self-reported via an interviewer-administered questionnaire [11]. Participants were only asked to provide occupational information if they reported having held a job outside of the home/farm for pay for more than one year. Participants were asked to report information about the longest job they had held during their life. For this job they were asked the job title and the frequency of performance of various tasks, including lifting/carrying/moving objects weighing > 10 lbs and heavy work while standing. Job titles were categorized into six industrial groups based on the 1990 Census of Population and Housing Alphabetical Index of Industries and Occupations: managerial and professional; technical, sales, and administrative support; service; farming, forestry, and fishing; precision production, craft, and repair; and operators, fabricators, and laborers [12]. In our analyses, jobs in the managerial and professional industry were used as the referent group. For occupational tasks, participants were asked to rate the frequency on a 5-point scale: 0 = never, 1 = seldom, 2 = sometimes, 3 = often, and 4 = always. In stratified analyses, occupational exposure categories were condensed due to decreased statistical power. Specifically, the frequency of lifting/carrying/moving tasks was condensed into three categories (never/seldom, sometimes, and often/always), the frequency of heavy work while standing was condensed into two categories (never/seldom and sometimes/often/always), and occupational industries were condensed into two categories: physical work (service; farming, forestry, and fishing; precision production, craft, and repair; and operators, fabricators, and laborers) and non-physical work (managerial and professional, and technical, sales, and administrative support).

### Outcomes

At the T2 time point, several outcome measures relevant to shoulder disability were collected. Participants were asked whether they had pain, aching, or stiffness (PAS) in the left (or right) shoulder on most days of any one month in the last year. If a participant answered “Yes” to this question for either the left or right shoulder they were counted as someone with prevalent shoulder symptoms. Additionally, they were asked to rate their shoulder symptoms as mild, moderate, or severe. A modified 9-item Disabilities Arm Shoulder and Hand (DASH) questionnaire was administered to assess upper extremity disability on a scale from 0 (no disability) to 100 (worst disability) [13]. These scores were further classified into five categories of disability (0, 1–25, 26–50, 51–75, 76–100). Finally, a back scratch test was used to assess shoulder function [14]. Participants were asked to reach over the right shoulder with the right hand while reaching with the left hand up the middle of the back to attempt to touch their fingers together. This measure was categorized into six categories: fingers touching or overlapping, measurable distance between extended middle fingers (1-14 cm, 15-21 cm, 22-29 cm, 30 + cm) or unable to perform the test. This test was then repeated on the opposite side with the left hand reaching over the left shoulder. The worst measure from the two sides was used for analyses.

### Other measurements

Other relevant variables collected by JoCoOA included self-reported sex (male/female), race (Black/White), and education (less than high school education/at least a high school education). Age was calculated based on self-reported birthdate. Height and weight were measured at both T1 and T2 follow-up visits allowing assessment of BMI and changes in BMI between follow-up time points. Data also included record of whether each participant was part of the original recruitment cohort, or the enrichment cohort.

### Statistical analysis

Descriptive statistics were calculated for the study population including demographic characteristics, distribution of occupational industries, and frequency of upper extremity occupational tasks. For evaluating associations of occupation industry and occupational tasks with prevalent shoulder symptoms, logistic regression models were used to calculate odds ratios and 95% confidence intervals as measures of association. For evaluating associations of occupation industry and occupational tasks with upper extremity disability, cumulative logit regression models, under the proportional odds (PO) assumption, were used to estimate associations with worse modified-DASH category and worse back scratch test category [15, 16]. The assumption of PO for these two polytomous outcomes was assessed using the Score test and, if significant at a 0.05 level, a partial PO model was assessed by testing for unequal slopes or effects across the number of levels of outcome, for each of the explanatory variables. If any of the contrast tests for a given variable was significant at 0.05, then that variable produced unequal slopes or effects for each level of outcome, otherwise the variable produced a proportional effect for worse outcome. For modified-DASH category a multinomial, partial PO model with cumulative logit regression was used, accounting for unequal slopes for covariates when indicated. For the back scratch test no evidence of violation of the PO assumption was found with the score test and a PO model was used with cumulative logit regression. All models were adjusted for age, sex, race, education, enrollment cohort, and time between follow-up visits T1 and T2. Primary analyses did not adjust for BMI as occupation may indirectly influence musculoskeletal problems through effects on obesity. Sensitivity analyses were run with adjustment for BMI to evaluate associations of occupational exposures independent of obesity. Associations stratified by sex and race, and corresponding interaction terms, were also calculated to evaluate effect measure modification. Interaction terms with a p-value < 0.10 were considered evidence of effect measure modification. For analyses of interaction terms, sensitivity analyses were run in which models additionally adjusted for length of time participants reported being employed in their ‘longest job held’, as differences in length of employment by sex or race might drive differences in associations.

### Ethics approval and informed consent

All participants completed informed consent forms. The Institutional Review Board at the University of North Carolina, Chapel Hill has continuously approved JoCoOA.

## Results

### Population selection and descriptive statistics

**Fig. 2:**
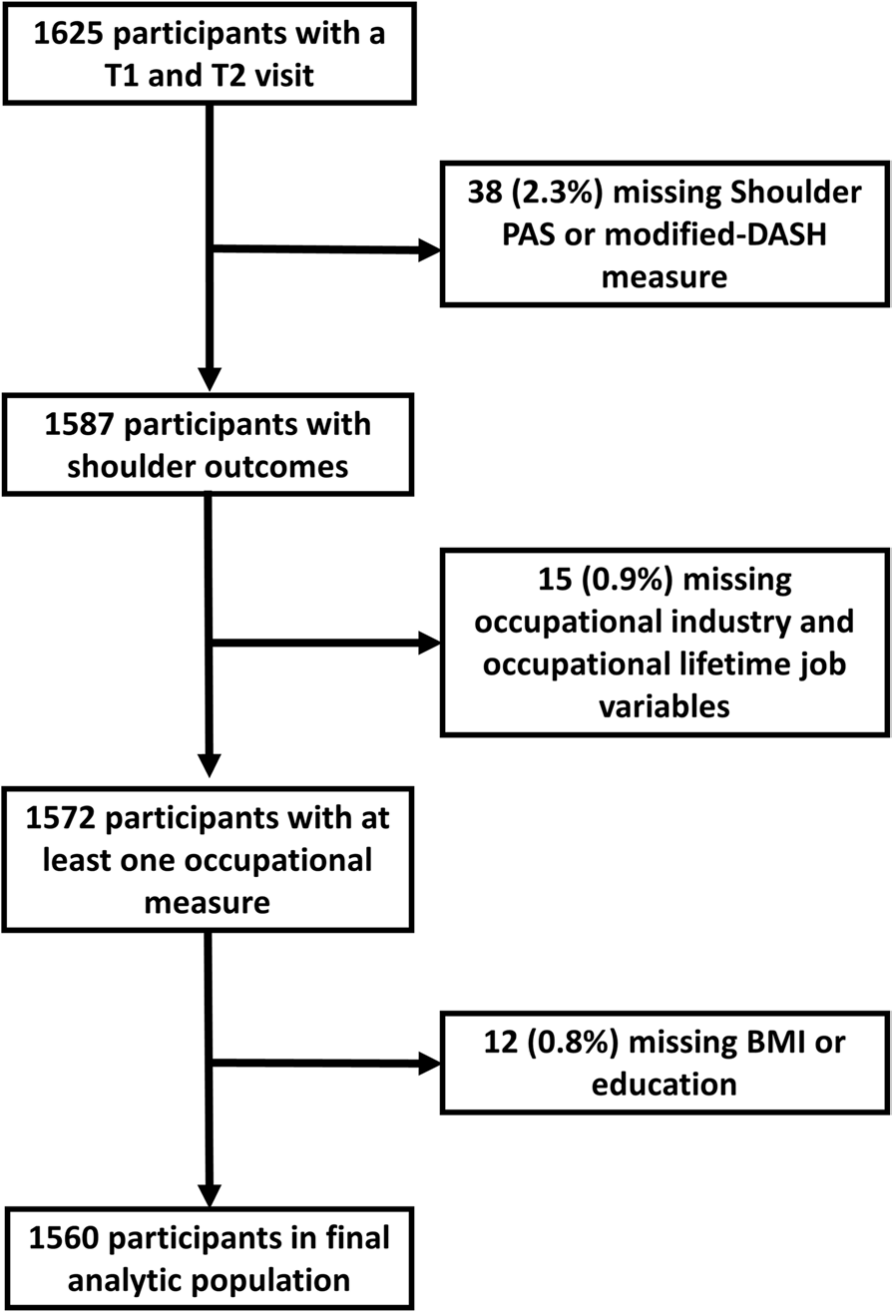
Flow chart of exclusion criteria applied to Johnston County Osteoarthritis Project participants to identify the analytic study population

Out of 1625 JoCoOA participants that attended both T1 and T2 follow-up visits, 38 (2.3%) did not have data available on shoulder symptoms or the modified-DASH measure (Fig. 2). An additional 15 participants did not report any occupational information, and 12 were missing information on either BMI or education. After all exclusions, 1560 participants (96% of participants with T1 and T2 visits) remained for inclusion in the final analytic population with an average of 6.5 years between T1 and T2 visits (range = 4–10 years).

In this population, 32% were men, 31% were Black, and the mean age at T1 visit was 62 years (standard deviation = 9 years) (Table 1). Among the 1487 people with information on occupational industry, 48% were in physical work industries (service; farming, forestry, and fishing; precision production, craft, and repair; and operators, fabricators, and laborers) (Table 1). Among the 1555 people reporting job requirements for lifting, carrying, or moving objects weighing > 10 lbs, 28% reported jobs that often required these tasks and 15% reported jobs that always required these tasks. Among the 1446 participants reporting job requirements for heavy work while standing, 7% reported jobs that often required these tasks and 3% reported jobs that always required these tasks.

**Table 1:** Baseline Characteristics of 1560 JoCoOA participants

Baseline Characteristics	n	%
Age in years at T1		
45–54	398	25.5
55–64	586	37.6
65–74	429	27.5
75 +	147	9.4
Men	505	32.4
Black race	476	30.5
Less than high school education	330	21.2
BMI group		
Underweight (< 18.5 kg/m^2^)	5	0.3
Healthy weight (18.5–24.9 kg/m^2^)	261	16.7
Overweight (25.0–29.9 kg/m^2^)	527	33.8
Obese (≥ 30.0 kg/m^2^)	767	49.2
BMI ≥ 5% increase from baseline to follow-up	499	32.0
Enrichment cohort	538	34.5
Occupational industry (missing = 73)		
1) Managerial and professional	345	22.1
2) Technical, sales, and administrative support	421	27.0
3) Operators, fabricators, and laborers	313	20.1
4) Service	212	13.6
5) Precision, production, craft, and repair	183	11.7
6) Farming, forestry, and fishing	13	0.8
Requires lifting, carrying, or moving objects weighing > 10 pounds (missing = 5)		
Never	203	13.0
Seldom	315	20.2
Sometimes	355	22.8
Often	443	28.4
Always	239	15.3
Requires heavy work while standing (missing = 114)		
Never	881	56.5
Seldom	228	14.6
Sometimes	183	11.7
Often	113	7.2
Always	41	2.6

*BMI* Body Mass Index

At the T2 follow-up visit, 24% of people reported shoulder PAS on most days for at least one month during the last year (Table 2). Among these 379 people, 3% reported severe symptoms, 11% reported moderate symptoms, and 10% reported mild symptoms. The median modified-DASH score was 2.78, representing minimal upper extremity disability. When DASH scores were categorized into five levels of disability, 1% of people reported the worst category of disability (unable to perform tasks, scores > 75), 6% reported severe difficulty performing tasks (scores > 50 and ≤ 75), and 15% reported moderate difficulty performing tasks (scores > 25 and ≤ 50). For the back scratch test, 5% of people had fingers touching or overlapping on at least one side, while 14% of people were unable to perform the test (Table 2). Among the 1350 people able to perform the back scratch test, the mean distance between fingers was 22 cm (standard deviation = 11.7 cm).

**Table 2:** Shoulder disability outcomes of interest at T2 Follow-up Visit for 1560 JoCoOA participants

	n^a^	%^a^
**Shoulder PAS on most days of any one month in the last year**	379	24.3
**Shoulder PAS maximum severity**		
None	1181	75.7
Mild	163	10.4
Moderate	173	11.1
Severe	43	2.8
**Modified-DASH score**^**a**^**, median (IQR)**	2.78	(0–22.2)
**Modified-DASH categories**		
0 (all items no difficulty)	666	42.7
> 0—≤ 25 (all items at worst some difficulty)	545	34.9
> 25—≤ 50 (all items at worst moderate difficulty)	227	14.6
> 50—≤ 75 (all items at worst severe difficulty)	100	6.4
> 75—≤ 100 (all items at worst unable)	22	1.4
**Back Scratch Shoulder Measure**^**b**^		
Fingers touch or overlap	73	4.7
Measurable distance between extended middle fingers 1–14 cm	269	17.2
Measurable distance between extended middle fingers 15–21 cm	346	22.2
Measurable distance between extended middle fingers 22–29 cm	334	21.4
Measurable distance between extended middle fingers 30 + cm	328	21.0
Unable to perform test	210	13.5

*PAS* Pain, aching, and stiffness, *DASH* Disabilities Arm Shoulder and Hand

^a^Modified DASH includes 9 items from the full DASH questionnaire: 1) Push open a heavy door, 2) Place an object on a shelf above your head, 3) Make a bed, 4) Change a lightbulb overhead, 5) Wash or blow dry your hair, 6) Wash your back, 7) Put on a pullover sweater, 8) Recreational activities in which you take some force or impact through your arm, shoulder, or hand (e.g. golf, hammering, tennis, etc.), and 9) Recreational activities in which you move your arm freely (e.g., playing Frisbee, badminton, etc.)

^b^Worst results from left/right shoulder

### Occupational exposure associations with measures of shoulder disability

Compared to workers in the managerial and professional industry, workers in both the operators/fabricators/laborers industry and service industry had statistically significantly worse outcomes for all measures of shoulder disability. Operators, fabricators, and laborers had 68% higher odds of reporting shoulder PAS, 71% higher odds of having a worse category of upper extremity disability as measured by the modified DASH score, and 137% higher odds of having a back scratch test measure in a worse category (Table 3). Service industry workers had 96% higher odds of reporting shoulder PAS, 123% higher odds of having a worse category of upper extremity disability as measured by the modified DASH score, and 127% higher odds of having a back scratch test measure in a worse category. Shoulder disability measures were also consistently worse in workers in the precision, production, craft, and repair industry, though the only statistically significant association was with worse back scratch test category (odds ratio [OR] = 1.68, 95%CI = 1.20–2.34).

**Table 3:** Associations between occupational exposures and shoulder disability measures

Exposure (modeled separately by exposure)	Any shoulder PAS	Worsening Modified-DASH category	Worsening back scratch test category
	OR (95% CI)	OR (95% CI)	OR (95% CI)
**Occupational industry, N = 1487**			
Managerial and professional (ref)	1.00	1.00	1.00
Technical, sales, and administrative support	1.12 (0.78, 1.60)	1.06 (0.81, 1.39)	1.12 (0.87, 1.44)
Operators, fabricators, and laborers	**1.68 (1.14, 2.48)**	**1.71 (1.25, 2.32)**	**2.37 (1.77, 3.18)**
Service	**1.96 (1.28, 3.01)**	**2.23 (1.57, 3.15)**	**2.27 (1.62, 3.17)**
Precision, production, craft, and repair	1.45 (0.93, 2.28)	1.43 (0.99, 2.04)	**1.68 (1.20, 2.34)**
Farming, forestry, and fishing	1.71 (0.50, 5.87)	0.88 (0.28, 2.70)	1.78 (0.63, 5.02)
**Requires lifting, carrying, or moving objects > 10 lbs, N = 1555**			
Never (ref)	1.00	1.00	1.00
Seldom	1.00 (0.64, 1.58)	0.87 (0.63, 1.22)	1.10 (0.80, 1.51)
Sometimes	1.33 (0.86, 2.05)	1.04 (0.75, 1.43)	1.08 (0.79, 1.48)
Often	**1.86 (1.22, 2.81)**	1.14 (0.84, 1.57)	1.30 (0.96, 1.77)
Always	**1.83 (1.15, 2.91)**	1.29 (0.90, 1.87)	1.33 (0.94, 1.89)
**Requires heavy work while standing, N = 1446**			
Never (ref)	1.00	1.00	1.00
Seldom	0.88 (0.60, 1.27)	0.93 (0.71, 1.23)	1.06 (0.82, 1.38)
Sometimes	**1.80 (1.26, 2.57)**	**1.39 (1.03, 1.89)**	**1.37 (1.03, 1.81)**
Often	1.25 (0.79, 1.98)	1.35 (0.94, 1.96)	1.21 (0.85, 1.73)
Always	**2.86 (1.48, 5.53)**	1.49 (0.79, 2.81)	1.74 (0.97, 3.11)

PAS *P*ain, aching, or stiffness, *DASH* Disability Arm Shoulder and Hand, *OR* Odds ratio, *CI* Confidence interval, *ref* reference category

Logistic regression model used for estimating associations with odds of any shoulder PAS. Multinomial, partial proportional odds model using cumulative logit regression to estimate associations with odds of having a worse category of the modified-DASH-9 score with unequal slopes for education in the first two exposure models, education and race for the third. Proportional odds model using cumulative logit regression to estimate associations with odds of having a worse category for the back scratch test. The six back scratch test categories from worst to best were: 1) unable to perform test, 2) fingers 30 + cm apart, 3) fingers 22–29 cm apart, 4) fingers 15–21 cm apart, 5) fingers < 15 cm apart, and 6) fingers overlapping or touching. All models adjusted for baseline cohort, age, sex, race, education, and time to follow-up. Statistically significant associations at alpha = 0.05 shown in **bold**

People with jobs that often or always required lifting/carrying/moving > 10 lb. objects had significantly higher odds of reporting shoulder PAS compared to people with jobs that never required lifting/carrying/moving > 10 lb. objects (ORs of 1.83 for ‘Always’ and 1.86 for ‘Often’, Table 3). People with jobs that often or always required listing/carrying/moving > 10 lb. objects also had worse modified-DASH scores and worse performance on the back scratch test, though these associations did not reach statistical significance. Jobs that seldom required lifting/carrying/moving > 10 lb. objects were not associated with any increases in shoulder disability outcomes.

People with jobs that sometimes or always required heavy work while standing had significantly higher odds of reporting shoulder PAS compared to people with jobs that never required heavy work while standing (ORs of 2.86 for ‘Always’ and 1.80 for ‘Sometimes’ Table 3). People with jobs that ‘Sometimes’ required heavy work while standing also had significantly worse modified-DASH scores and worse performance on the back scratch test compared to people with jobs that never required heavy work while standing (modified-DASH OR = 1.39, 95%CI = 1.03–1.89; back scratch test OR = 1.37, 95%CI = 1.03–1.81). Jobs that ‘Always’ required heavy work while standing were associated with even higher odds of worse modified-DASH scores and back scratch test performance, but these associations were not statistically significant as these estimates were less precise (modified-DASH OR = 1.49, 95%CI = 0.79–2.81; back scratch test OR = 1.74, 95%CI = 0.97–3.11). Jobs that seldom required heavy work while standing were not associated with any increases in shoulder disability outcomes.

Results were similar in sensitivity analysis that additionally adjusted for BMI at the T1 time point and for change in BMI from the T1 to T2 time points (Table 4).

**Table 4:** Associations between occupational exposures and shoulder outcomes after additionally adjusting for body mass index

Exposure (modeled separately by exposure)	Any shoulder PAS	Worsening Modified-DASH category	Worsening back scratch test category
	OR (95% CI)	OR (95% CI)	OR (95% CI)
**Occupational industry, N = 1487**			
Managerial and professional (ref)	1.00	1.00	1.00
Technical, sales, and administrative support	1.11 (0.77, 1.59)	1.03 (0.79, 1.35)	1.05 (0.82, 1.35)
Operators, fabricators, and laborers	**1.62 (1.10, 2.40)**	**1.57 (1.15, 2.14)**	**2.18 (1.62, 2.92)**
Service	**1.87 (1.21, 2.89)**	**1.98 (1.40, 2.81)**	**1.82 (1.29, 2.56)**
Precision, production, craft, and repair	1.40 (0.89, 2.21)	1.33 (0.92, 1.90)	**1.41 (1.01, 1.96)**
Farming, forestry, and fishing	1.75 (0.51, 6.04)	0.90 (0.29, 2.76)	1.99 (0.72, 5.54)
**Requires lifting, carrying, or moving objects > 10 lbs, N = 1555**			
Never (ref)	1.00	1.00	1.00
Seldom	1.02 (0.64, 1.61)	0.92 (0.66, 1.28)	1.14 (0.82, 1.58)
Sometimes	1.32 (0.86, 2.05)	1.04 (0.75, 1.43)	1.04 (0.76, 1.43)
Often	**1.84 (1.21, 2.80)**	1.13 (0.82, 1.55)	1.24 (0.91, 1.69)
Always	**1.78 (1.12, 2.85)**	1.26 (0.87, 1.83)	1.26 (0.88, 1.79)
**Requires heavy work while standing, N = 1446**			
Never (ref)	1.00	1.00	1.00
Seldom	0.88 (0.61, 1.29)	0.96 (0.73, 1.27)	1.13 (0.87, 1.46)
Sometimes	**1.83 (1.28, 2.62)**	**1.40 (1.03, 1.90)**	**1.32 (1.00, 1.75)**
Often	1.20 (0.75, 1.91)	1.25 (0.86, 1.82)	0.96 (0.68, 1.37)
Always	**2.65 (1.37, 5.14)**	0.73, 2.63)	1.49 (0.85, 2.63)

*PAS* Pain, aching, or stiffness, *DASH* Disability Arm Shoulder and Hand, *OR* Odds ratio, *CI* Confidence interval, *ref* reference category

Logistic regression model used for estimating associations with odds of any shoulder PAS. Multinomial, partial proportional odds model using cumulative logit regression to estimate associations with odds of having a worse category of the modified-DASH-9 score with unequal slopes for education in the first two exposure models, education and race for the third. Proportional odds model using cumulative logit regression was used to estimate associations with odds of having a worse category for the back scratch test. The six back scratch test categories from worst to best were: 1) unable to perform test, 2) fingers 30 + cm apart, 3) fingers 22–29 cm apart, 4) fingers 15–21 cm apart, 5) fingers < 15 cm apart, and 6) fingers overlapping or touching. All models adjusted for baseline cohort, age, sex, race, education, time to follow-up, baseline BMI, and 5% increase in BMI from baseline to follow-up. Statistically significant associations at alpha = 0.05 shown in **bold**

### Associations stratified by sex and race

Employment in a physical work industry was associated with worse shoulder disability for both men and women across all outcome measures (Table 5). Interaction terms between sex and occupational industry did not indicate effect measure modification. Jobs that ‘often/always’ required lifting/carrying/moving > 10 lb. objects and jobs that sometimes/often/always required heavy work while standing were significantly associated with higher odds of shoulder PAS in both men and women. While the magnitude of the associations was larger in men, interaction terms did not indicate effect modification. Jobs that often/always required lifting/carrying/moving > 10 lb. objects and jobs that sometimes/often/always required heavy work while standing were significantly associated with higher odds of worse modified-DASH score category among men, but not women. This corresponded with a significant interaction between sex and frequency of heavy work while standing (interaction p-value = 0.06). However, the interaction term for sex and frequency of lifting/carrying/moving was not significant (interaction p-value = 0.34). For the back scratch test, no interactions with sex were identified.

**Table 5:** Associations between occupational exposures and shoulder disability measures by sex

	Any shoulder PAS		Worsening Modified-DASH category		Worsening back scratch test category	
Exposure (modeled separately by exposure)	Women	Men	Women	Men	Women	Men
	OR (95% CI)	OR (95% CI)	OR (95% CI)	OR (95% CI)	OR (95% CI)	OR (95% CI)
**Physical work occupational industry, N = 1487**	**1.51** **(1.10, 2.07)**	**1.78** **(1.13, 2.80)**	**1.52** **(1.18, 1.96)**	**2.11** **(1.47, 3.04)**	**2.16** **(1.69, 2.77)**	**1.68** **(1.20, 2.34)**
**Requires lifting, carrying, or moving objects weighing > 10 lbs, N = 1555**						
Never/Seldom (ref)	1.00	1.00	1.00	1.00	1.00	1.00
Sometimes	1.40 (0.96, 2.04)	1.12 (0.54, 2.32)	1.14 (0.86, 1.52)	1.10 (0.65, 1.89)	0.95 (0.72, 1.26)	1.24 (0.76, 2.00)
Often/Always	**1.78** **(1.28, 2.48)**	**1.94** **(1.10, 3.43)**	1.18 (0.91, 1.53)	**1.59** **(1.03, 2.45)**	1.27 (0.99, 1.64)	1.20 (0.81, 1.79)
**Requires heavy work while standing sometimes / often / always, N = 1446**	**1.45** **(1.01, 2.09)**	**2.30** **(1.47, 3.59)**	1.17 (0.86, 1.58)	**1.87** **(1.29, 2.71)**	**1.34** **(1.00, 1.78)**	1.31 (0.93, 1.85)

Logistic regression model used for estimating associations with odds of any shoulder PAS and odds of being unable to perform the back scratch trial. Multinomial, partial proportional odds model using cumulative logit regression to estimate associations with odds of having a worse category of the modified-DASH-9 score with unequal slopes for education. Proportional odds model using cumulative logit regression was used to estimate associations with odds of having a worse category for the back scratch test. The six back scratch test categories were: 1) unable to perform test, 2) fingers 30 + cm apart, 3) fingers 22–29 cm apart, 4) fingers 15–21 cm apart, 5) fingers <15 cm apart, and 6) fingers overlapping or touching. All models adjusted for baseline cohort, age, race, education, and time to follow-up. Significant associations at alpha = 0.05 shown in **bold.** Effect underlined if exposure effect is statistically different by sex effect modifier with p-value < 0.1 for the interaction term

*PAS* Pain, aching, or stiffness, *DASH* Disability Arm Shoulder and Hand, *OR* Odds ratio, *CI* Confidence interval, *ref* reference category

Employment in a physical work industry was associated with worse shoulder disability for both Black and White participants across all outcome measures (Table 6), and interaction terms did not indicate effect measure modification. Similarly, no significant interactions were observed between race and job requirements for lifting/carrying/moving > 10 lb. objects, but the magnitude of associations did differ. For instance, among White participants, jobs that ‘sometimes’ or ‘often/always’ required lifting/carrying/moving > 10 lb. objects were associated with 52% higher odds and 96% higher odds of shoulder PAS, respectively, when compared to people in jobs that ‘never/rarely’ required those tasks. Among Black participants, no association was observed with jobs that ‘sometimes’ required lifting/carrying/moving > 10 lb. objects (OR = 0.99), while jobs that ‘often/always’ required lifting/carrying/moving > 10 lb. objects were only associated with 61% higher odds of shoulder PAS. Conversely, for the modified-DASH measure, jobs that ‘often/always’ required lifting/carrying/moving > 10 lb. objects had a stronger association with higher odds among Black participants than White participants (Table 6). Jobs that sometimes/often/always required heavy work while standing were significantly associated with higher odds of shoulder PAS in White participants, but not among Black participants. This corresponded with a significant interaction between race and frequency of heavy work while standing (interaction p-value = 0.05). For the back scratch test, no interactions with race were identified.

**Table 6:** Associations between occupational exposures and shoulder disability measures by race

	Any shoulder PAS		Worsening Modified-DASH category		Worsening back scratch test category	
Exposure (modeled separately by exposure)	White	Black	White	Black	White	Black
	OR (95% CI)	OR (95% CI)	OR (95% CI)	OR (95% CI)	OR (95% CI)	OR (95% CI)
**Physical work occupational industry, N = 1487**	**1.46** **(1.08, 1.99)**	**2.07** **(1.19, 3.60)**	**1.74** **(1.36, 2.23)**	**1.52** **(1.02, 2.28)**	**2.02** **(1.59, 2.56)**	**1.90** **(1.31, 2.74)**
**Requires lifting, carrying, or moving objects weighing > 10 lbs, N = 1555**						
Never/Seldom (ref)	1.00	1.00	1.00	1.00	1.00	1.00
Sometimes	**1.52** **(1.01, 2.27)**	0.99 (0.54, 1.80)	1.15 (0.85, 1.57)	1.07 (0.68, 1.69)	1.09 (0.81, 1.46)	0.90 (0.59, 1.37)
Often/Always	**1.96** **(1.39, 2.77)**	1.61 (0.98, 2.64)	1.16 (0.89, 1.50)	**1.71** **(1.14, 2.55)**	1.22 (0.95, 1.56)	1.29 (0.88, 1.87)
**Requires heavy work while standing sometimes / often / always, N = 1446**	**2.13** **(1.52, 2.99)**	1.16 (0.72, 1.89)	**1.42** **(1.07, 1.90)**	1.39 (0.93, 2.08)	**1.32** **(1.01, 1.73)**	1.34 (0.92, 1.95)

Logistic regression model used for estimating associations with odds of any shoulder PAS and odds of being unable to perform the back scratch trial. Multinomial, partial proportional odds model using cumulative logit regression to estimate associations with odds of having a worse category of the modified-DASH-9 score with unequal slopes for education. Proportional odds model using cumulative logit regression was used to estimate associations with odds of having a worse category for the back scratch test. The six back scratch test categories were: 1) unable to perform test, 2) fingers 30 + cm apart, 3) fingers 22–29 cm apart, 4) fingers 15–21 cm apart, 5) fingers <15 cm apart, and 6) fingers overlapping or touching.  All models adjusted for baseline cohort, age, sex, education, and time to follow-up. Significant associations at alpha = 0.05 shown in **bold.** Effect underlined if exposure effect is statistically different by race effect modifier with p-value < 0.1 for the interaction term

*PAS* Pain, aching, or stiffness, *DASH* Disability Arm Shoulder and Hand, *OR* Odds ratio, *CI* Confidence interval, *ref* reference category

Results were similar in sensitivity analyses that additionally adjusted for the length of employment for “longest job held” with the same statistically significant interaction terms identified.

## Discussion

Function of the upper extremities, and specifically the shoulder, is essential to daily activities in both the home and the workplace. Occupational burdens are likely key contributors to the development of shoulder injuries and pathology that leads to pain and disability. In the JoCoOA, we found that compared to people employed in the managerial/professional industry, people in industries with physically demanding jobs had higher odds of both shoulder symptoms and worse upper extremity disability, particularly the service industry and operators, fabricators, and laborers. When examining specific occupational requirements, jobs that often or always required lifting/moving > 10 lb. objects and jobs that required heavy work while standing were associated with higher odds of shoulder symptoms. The association between heavy work while standing and shoulder symptoms was stronger among men and White workers. But overall, physically demanding occupations were associated with higher prevalence of shoulder pain and disability across populations, regardless of race or sex.

Consistent with our results, several European studies have shown that occupational upper extremity loads are associated with risk of shoulder pain and shoulder disorders broadly [2–4, 17–19]. Recently, studies have started to identify occupational risk factors for specific shoulder disorders [20–22]. This includes work from our research team showing a doubling of risk for rotator cuff disease surgery with long-term exposure to physical work exposures in the UK [20]. In Denmark, an investigation of the relationships between cumulative occupational shoulder exposures and different diagnoses related to shoulder impingement surgery found particularly strong associations for patients with osteoarthritis diagnoses, including a doubling of risk for workers for long-term exposure to tasks requiring upper arm-elevation or repetitive shoulder movements [22]. A couple US studies have also provided preliminary evidence of associations between physical work exposures and shoulder disorders, though these studies had limited statistical power due to small sample sizes (case Ns of 55 and 18) [23, 24].

Some studies have also evaluated sex-specific associations with mixed results. Another study by the Danish research group reported no differences in sex-specific associations of occupational exposures on subacromial impingement surgery [7]. But an earlier Finnish study demonstrated differences by sex in the associations of specific occupational tasks with chronic shoulder disorders [2]. For instance, lifting heavy loads had a stronger association with shoulder disorders among women, while repetitive movements had a stronger association with shoulder disorders among men [2]. In our study, the only significant difference in sex-stratified results was the association of heavy work while standing with shoulder function, which was significantly stronger in men. Differences in these associations may be due to the limited specificity of this measure. While we accounted for differences in length of time employed in the jobs reported, there may also be differences in the specific physical tasks being done. For instance, men may be doing more strenuous or more repetitive heavy work on average.

As prior large studies of the relationship between occupational exposures and risk of shoulder symptoms and disability have been conducted in European populations, these populations have all been predominantly White. And the few existing US occupational studies of shoulder disorders did not have sufficient sample sizes for evaluating associations by race [23, 24]. By contrast, the JoCoOA population was recruited in the United States with a population-based sampling design that provided a large, diverse, representative sample of a mostly rural region of North Carolina in which > 30% of the population was Black. This allowed sufficient statistical power to estimate and compare race-stratified associations, which have not been evaluated in prior studies. In our study, most associations did not differ by race, but we did observe a stronger association between heavy work while standing and shoulder symptoms in White than Black participants. Similar to the effect measure modification observed by sex, these results may be explained by differences in the type of heavy work conducted. Of note, a prior JoCoOA study demonstrated that shoulder symptoms did not differ by sex or race overall [8], so differences in the relative odds do not appear to be driven by differences in the baseline prevalence between groups.

There were several limitations to this study. First, occupational measures were self-reported and it is possible that those who experienced upper extremity pain or disability may have over-reported the frequency of physical tasks at work. However, a prior study comparing self-reported upper extremity exposures to direct observation did not demonstrate such a bias among people with musculoskeletal symptoms [25]. Furthermore, it is less likely that occupational industry would be reported differentially based on symptoms or disability. We also did not have measures of shoulder-specific occupational exposures, such as arm elevation, which could be more strongly associated with shoulder disability than the general measures of lifting/moving objects and heavy work. Second, shoulder disability measures were only available at a single time point, so we could not evaluate incident disability or whether symptoms or function worsened over time while employed in a physically demanding job. Relatedly, if some participants had endured chronic shoulder symptoms for many years prior to our occupational assessment, this may have reduced the likelihood that they remained in a physically demanding job. If such a bias is present, then the true effects of occupational exposures on shoulder disability may be even stronger than the associations reported here. JoCoOA participants also did not have clinical examinations of their shoulders and so we cannot identify associations with particular shoulder injuries or pathologies. Prior evidence indicates that occupational shoulder demands may influence a myriad of shoulder disorders, including tendonitis, rotator cuff tears, and osteoarthritis [20, 22]. Finally, as we did not have lifetime occupational histories we could not evaluate the cumulative impact of occupational exposures over time, and some differences in effects by sex and race may be driven by differences in total years of exposure. Given the older age of our study population, most participants have likely had numerous jobs over time.

In conclusion, we found that having a job in a physical work industry, or a job that specifically requires physically demanding tasks is associated with increased occurrence of shoulder pain and disability. While some associations differed by race or sex, significant associations between physical work and shoulder disability measures were observed across all populations that were examined. Mitigating specific physical work demands may reduce shoulder-related disability. Future prospective studies that capture more detailed occupational exposures along with follow-up for incident shoulder pain and disability will be key to informing appropriate mitigation measures. For example, devices such as exoskeletons are being developed to reduce biomechanical loading on the shoulder joint [26]. Evaluation of interventions such as these will be important to determine ways to reduce shoulder disability in the future.

## Data Availability

The data that support the findings of this study are available on request from the study authors. The data are not publicly available due to privacy or ethical restrictions.

## Abbreviations

BMI: Body mass index
CI: Confidence interval
DASH: Disabilities Arm Shoulder and Hand
JoCoOA: Johnston County Osteoarthritis Project
OA: Osteoarthritis
OR: Odds ratio
PAS: Pain, aching, or stiffness
T1: First follow-up visit
T2: Second follow-up visit
